# Engineering collagenous analogs of connective tissue extracellular matrix

**DOI:** 10.3389/fbioe.2022.925838

**Published:** 2022-10-14

**Authors:** Philip A. P. Brudnicki, Matthew A. Gonsalves, Stephen M. Spinella, Laura J. Kaufman, Helen H. Lu

**Affiliations:** ^1^ Biomaterials and Interface Tissue Engineering Laboratory, Department of Biomedical Engineering, Columbia University, New York, NY, United States; ^2^ Department of Chemistry, Columbia University, New York, NY, United States

**Keywords:** ECM model, collagen, biofabrication, green electrospinning, glutaraldehyde, crosslinking

## Abstract

Connective tissue extracellular matrix (ECM) consists of an interwoven network of contiguous collagen fibers that regulate cell activity, direct biological function, and guide tissue homeostasis throughout life. Recently, ECM analogs have emerged as a unique *ex vivo* culture platform for studying healthy and diseased tissues and in the latter, enabling the screening for and development of therapeutic regimen. Since these tissue models can mitigate the concern that observations from animal models do not always translate clinically, the design and production of a collagenous ECM analogue with relevant chemistry and nano- to micro-scale architecture remains a frontier challenge in the field. Therefore, the objectives of this study are two-fold— first, to apply green electrospinning approaches to the fabrication of an ECM analog with nanoscale mimicry and second, to systematically optimize collagen crosslinking in order to produce a stable, collagen-like substrate with continuous fibrous architecture that supports human cell culture and phenotypic expression. Specifically, the “green” electrospinning solvent acetic acid was evaluated for biofabrication of gelatin-based meshes, followed by the optimization of glutaraldehyde (GTA) crosslinking under controlled ambient conditions. These efforts led to the production of a collagen-like mesh with nano- and micro-scale cues, fibrous continuity with little batch-to-batch variability, and proven stability in both dry and wet conditions. Moreover, the as-fabricated mesh architecture and native chemistry were preserved with augmented mechanical properties. These meshes supported the *in vitro* expansion of stem cells and the production of a mineralized matrix by human osteoblast-like cells. Collectively these findings demonstrate the potential of green fabrication in the production of a collagen-like ECM analog with physiological relevance. Future studies will explore the potential of this high-fidelity platform for elucidating cell-matrix interactions and their relevance in connective tissue healing.

## Introduction

The extracellular matrix (ECM) is a diverse network of biopolymers that are organized and dynamically remodeled to meet the demands of physiological function. It can consist of over 300 different proteins, including over 20 types of collagens, several dozen proteoglycan and approximately 200 glycoproteins. (Frantz et al., 2010; Hynes and Naba, 2012; Theocharis et al., 2016). Each of these proteins may be organized with controlled interactions to impart structural and mechanical properties that are specific for the tissue of interest. Given this vast complexity, it is not surprising that a wide range of diseases can arise from disruption to the ECM’s structure and composition. These include various cancers (Muschler and Streuli, 2010; Pickup et al., 2014), fibroproliferative diseases (Poli and Parola, 1997; Friedman, 2008), and many debilitating orthopaedic conditions, such as osteoporosis, osteoarthritis, chondrodysplasias, and intervertebral disk degeneration (Alberts et al., 2002; Parvizi, 2010). To better understand and treat these diverse diseases and conditions, an accurate model of the ECM is necessary to conduct physiologically relevant studies *in vitro*.

**FIGURE 1:**
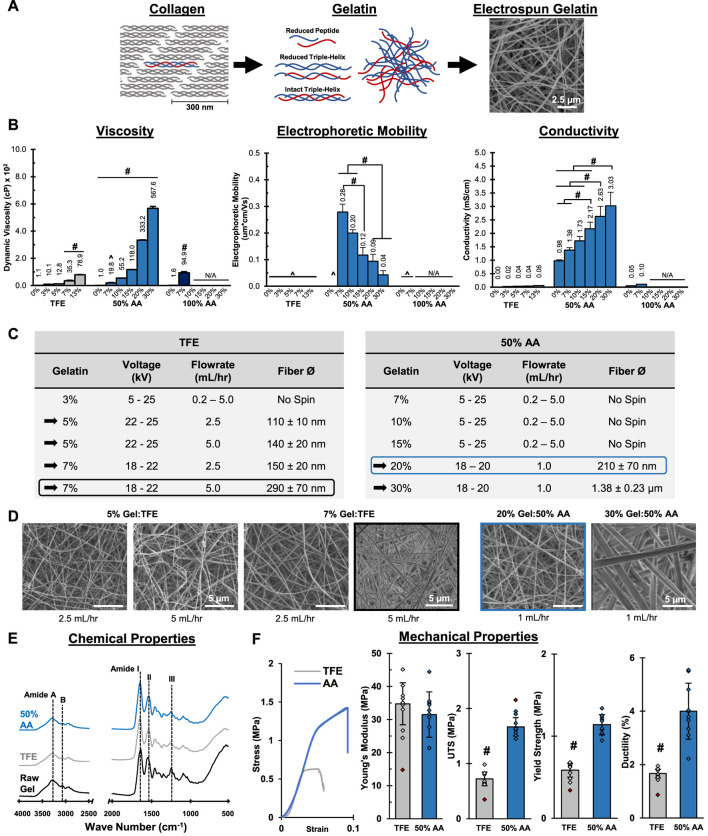
**Mesh Fabrication: Solvent Optimization and Electrospinning.** 50% acetic acid (AA) dissolves gelatin and supports mesh fabrication by electrospinning. The resulting mesh morphology, chemical composition and mechanical properties are comparable to meshes prepared in trifluoroethanol (TFE). **(A)** A schematic representing the structure of gelatin and its relationship to collagen. **(B)** Dynamic viscosity, electrophoretic mobility and conductivity measurements for gelatin solutions (*n* = 3). ^= Below Limit of Detection for Instrument. N/A = Not available due to sample gelling before analysis. **(C)** Successful electrospinning parameters using TFE and 50% AA. **(D)** Fiber morphology and diameter (*n* = 50) for meshes prepared from stable electrospins identified in part c. (SEM. 5,000x). **(E)** Chemical composition (FTIR-ATR). **(F)** Mesh tensile properties (*n* = 10). Red markers indicate sample outliers. #=*p* < 0.05.

Among the proteins of the ECM, the most abundant are collagens, which make up over 30% of the total protein mass in the human body (Frantz et al., 2010; Fratzl, 2008). Among collagens, the most prevalent is type I, which is found in many connective and load-bearing tissues such as skin, tendons, ligaments, and bone (Vanderby and Provenzano, 2003). Collagen has a unique, hierarchical structure that provides mechanical support while also promoting cell attachment and activity through its chemistry. This structure is achieved through its triple-helical domains that are formed from three polypeptide chains (Figure 1) containing the amino acid sequence Glycine-X-Y, where X and Y are commonly proline and hydroxyproline (Shoulders and Raines, 2009). The hydroxyl groups of hydroxyproline assist in hydrogen bonding and enable interactions between adjacent peptides and fibrils, further building and stabilizing higher order collagen structures (Xu et al., 2019). The cyclic structure of these amino acids is partially responsible for driving the helical conformation of the tropocollagen monomer via steric hindrance (Shoulders and Raines, 2009; Ricard-Blum, 2011). Following this, tropocollagen molecules self-assemble into microfibril structures that are approximately 5 nm in diameter and several hundred nanometer long, which then further assemble into large fiber structures that are several hundred nanometer in diameter and several centimeter long. This final step, which is critical for creating collagen’s high resilience to mechanical strain, is achieved through interchain crosslinks, which represent chemical or physical bonding between fibrils and most frequently from disulfide bond formation, transglutaminase conjugation of glutamine-lysine, lysyl oxidase crosslinking, and glycation (Siegel et al., 1970; Esposito and Caputo, 2005; Karsdal, 2019). While collagen fibrils themselves are not viscoelastic, through a slipping motion between the ordered fibrous structure, they can exhibit high levels of tissue deformation, which is a key characteristic of many connective tissues (Lorenzo and Caffarena, 2005; Shen et al., 2011). Beyond providing structural stability and supporting mechanotransduction and cell signaling throughout the ECM, collagen is also rich with many binding domains that support cell adhesion. It is decorated with ligands compatible with the many cell-surface integrins and discoidin domain receptors that support cell attachment, forming the critical connection between cells and the ECM (Theocharis et al., 2016). Given its high prevalence and unique chemical and physical properties, it is clear that collagen is a vital component of the ECM and is a critical feature when developing a bioactive analog of connective tissue ECM.

Despite the significant role collagen plays in cell-matrix interaction, the majority of *in vitro* studies rely on non-physiologic culturing substrates such as tissue culture polystyrene (TCPS) (Altering, 1966; Zeiger et al., 2013; Lerman et al., 2018). Since its adaption for eukaryotic cell culture in 1966, TCPS has been the standard platform for *in vitro* culture. Polystyrene is easy to synthesize, reproducible at scale, and its surface chemistry may be readily modified to accommodate a plethora of cell types. However, the chemical, structural, and mechanical properties of TCPS are radically different from collagenous ECM. Being a 2D substrate with a modulus on the scale of gigapascals, TCPS is orders of magnitude stiffer than all but the hardest of human tissues, such as bone and dentin (Kinney et al., 2003; Guimarães et al., 2020). Thus not surprisingly, long term stem cell culture on TCPS results in loss of stemness and drives these cells towards the osteogenic lineage (Tatullo et al., 2016; Gerardo et al., 2019). Similarly, the deleterious effect of cell passaging on phenotypic expression may be largely attributed to extended exposure to TCPS during *ex vivo* expansion (Gerardo et al., 2019; Rao et al., 2019). Furthermore, the lack of a fibrillar architecture coupled with the inherent difference in chemistry between TCPS and the native ECM at best attenuates, and at its worst, alters cell response beyond the realm of physiological relevance. Surface functionalization by coating with collagen or other ECM proteins (e.g., fibronectin) have mitigated these concerns but are costly and still result in cells being exposed to static and non-representative doses of the biomolecule (Lerman et al., 2018). These limitations significantly limit the applicability of TCPS and underscore the need for better models of the ECM.

Given the critical role of collagen architecture in directing cell response, significant focus has been directed towards the design and fabrication of ECM analogues with biomimetic organization (Lutolf and Hubbell, 2005; Moffat et al., 2009). To this end, electrospinning has been explored extensively for the generation of nano- and micro-scale fiber mesh using a variety of synthetic and biopolymers (Li et al., 2002; Ma et al., 2005; Sell et al., 2007; Zhang et al., 2007; Subramony et al., 2013; Lee et al., 2017). In electrospinning, electrostatic forces are used to destabilize polymer solutions and cause the ejection of fine polymer strands that are then collected into a fibrous mat (Reneker and Chun, 1996; Reneker et al., 2000). These nano- and micro-fibrous substrates exhibit biomimetic architectures (diameter, alignment) that can be controlled at physiologically relevant scales, making electrospinning an ideal method for producing ECM analogues and targeting desired tissue architectures or states (Lee et al., 2017). As such, similar to TCPS, the collagen chemistry and bioactive domains that readily regulate and guide cell function are missing from these synethic, polymer-based systems. Alternatively, biological and synthetic polymer blends have also been explored (Jose et al., 2009; Kriegel et al., 2009). Here, the biopolymers introduce the biochemical features of the ECM to the analog, improving the system’s biomimicry. Unfortunately, these systems still rely on synthetic materials to provide structural support and sufficient mechanical properties, which ultimately confound results when studying cell activity. Surface coatings and incorporation of growth factors (Chew et al., 2005; Boushell et al., 2019; Qu et al., 2019) have mitigated some of these concerns for regenerative medicine applications, but remain limited in scope and biological relevance as ECM analogues.

To better mimic the matrix composition, Matrigel^®^, consisting of a mixture of basement membrane proteins isolated from murine Englebreth-Holm-Swarm tumors, is commonly used (Kleinman et al., 1982). While it retains the natural basement membrane structure and composition after isolation *in vitro*, being derived from tumors, there is significant batch-to-batch variability and the isolated matrix often contains growth factors at non-physiologic concentrations and combinations (Vukicevic et al., 1992). Moreover, with a relatively low collagen I and high collagen IV content, compositionally it does not match the predominantly collagen I matrix intrinsic to connective tissues. While collagen-based hydrogels have also been used extensively for *in vitro* culture, their structural and mechanical properties are sub-physiologic, lacking both continuous fiber morphology and extended organization of the native ECM (Nöth et al., 2005; Oryan et al., 2018).

The feasibility of electrospinning collagen has also been explored, but largely with gelatin given the high cost of and difficulty solubilizing pure collagen (Huang et al., 2004; Zhang et al., 2005a). Gelatin is produced by hydrolyzing collagen, which breaks down its tertiary structure, yielding a heterogeneous polypeptide solution (Figure 1) with enhanced solubility (Harrington and Von Hippel, 1962). Most importantly, along with composition, it retains collagen’s primary and secondary structure, which is rich with cell-binding motifs that support cell-matrix interactions (Ratanavaraporn et al., 2006; Davidenko et al., 2016). Furthermore, it is readily isolated from porcine and bovine connective tissues and thus inexpensive and easily obtainable. However, irrespective of the base material used, one drawback of using electrospinning to prepare the ECM analog is that it typically relies on harsh chemical solvents like trifluoroethanol (TFE), hexafluoroisopropanol, and dichloromethane. These solvents reduce substrate bioactivity and moreover, are associated with negative health and ecological effects, making them obstacles to large scale mesh production (Persano et al., 2013; Impurities, 2018). Recently, Mosher et al. (2021) identified the FDA Q3C solvent, acetic acid (AA) as a more biocompatible and “green” solvent for biofabrication. Thus, the first objective of this study is to optimize fabrication conditions using gelatin and AA as the solvent for the production of a fibrous collagen-like mesh.

**FIGURE 2:**
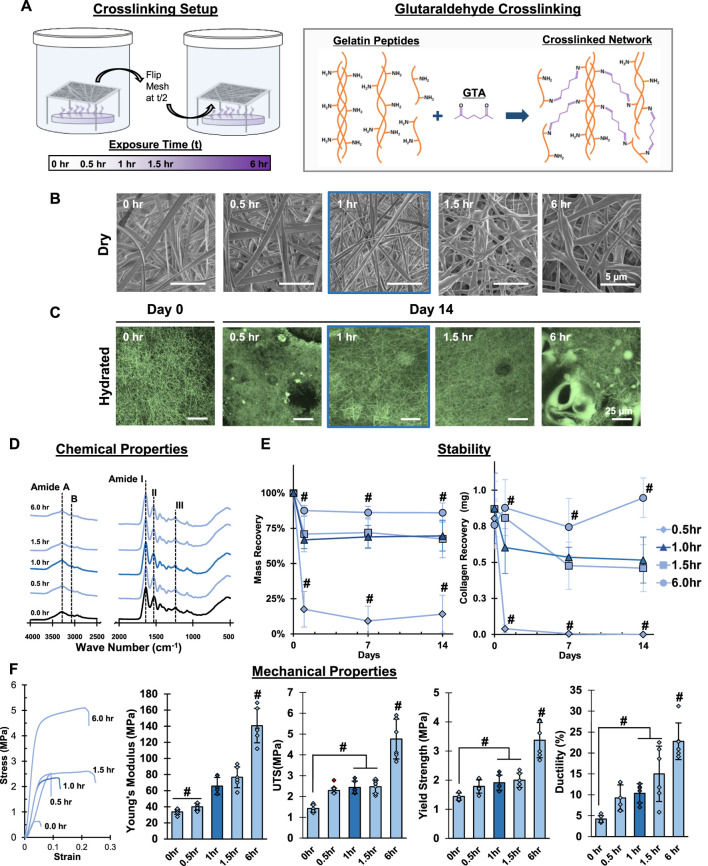
**Mesh Fabrication: Crosslinking Duration.** Glutaraldehyde exposure for 1 hour stabilizes meshes and enhances tensile properties without changing fiber morphology or chemistry. **(A)** Schematic of the crosslinking procedure and chemical reaction. **(B)** Dry morphology (SEM, 5,000x) and **(C)** hydrated morphology (CRM, 100x) of meshes after crosslinking. **(D)** Chemical composition (FTIR-ATR). **(E)** Mesh stability by mass recovery and gelatin recovery after incubation at 37°C in cell culture medium (14 days, *n* = 5). **(F)** Mesh tensile properties (*n* = 10). Red marker indicates outliers. #=*p* < 0.05.

Additional challenges in the production of collagen analogs include the fact that gelatin meshes require chemical stabilization or crosslinking following fabrication. Glutaraldehyde (GTA) is the most commonly used crosslinking agent, with highly reactive dialdehyde groups that readily bind to amines that are abundant in the amino acids that make up collagen’s peptides (Bigi et al., 2001; Farris et al., 2010). Thus, adjacent gelatin fibers can be crosslinked by GTA, ultimately stabilizing the mesh architecture in aqueous environments (Figure 2A). However, GTA reactions are extremely difficult to control, resulting in highly variable mechanical properties and physical features between batches, resulting in a wide range of methods and approaches reported in literature (Zhang et al., 2006; Ju et al., 2010; Li et al., 2016). When applied in liquid form, GTA can partially or fully obliviate the fibrous architecture of the mesh during the crosslinking, resulting in flat sheets instead. While vapor crosslinking is better at preserving 3D fiber organization, vapor formation and interaction with mesh fibers is extremely difficult to control, being highly dependent on experimental setup and environmental conditions. Thus, the second objective of this study is to identify critical factors that impact GTA vapor crosslinking and determine the optimal parameters that will stabilize gelatin meshes without altering their biomimetic structure and chemical composition, all while remaining biocompatible.

This study aims to develop and standardize a method for fabricating a biomimetic collagenous ECM-like substrate. To this end, this ECM analog will be collagen-based, biocompatible, consist of continuous fibers, demonstrate both viscoelastic and elastic behavior, exhibit relevant mechanical properties, and remain stable for at least 14 days under cell culture conditions. After mesh development and characterization, biocompatibility will be tested using both cell-line and primary culture. It is anticipated that a high-fidelity collagen-based ECM analog will be fabricated under optimal electrospinning and crosslinking conditions, with physiologically relevant structural and mechanical properties reminiscent of the native collagenous ECM.

## Materials and methods

### Mesh fabrication

Gelatin (porcine-derived, ∼300 g bloom, type A) was dissolved in trifluoroethanol (TFE), 50% acetic acid (AA, prepared in deionized water) or glacial (100%) AA. Gelatin was solubilized in TFE (3, 5, 7, and 13% w/v), 50% AA (7, 10, 15, 20, and 30% w/v), and glacial AA (7, 10, 15, 20, and 30% w/v) by mixing at 45°C for up to 20 min (Fischer Brand Analog Vortex, setting 7) to accelerate dissolution. If the gelatin was not dissolved or had solidified after 20 min, the solution was considered not feasible for electrospinning. Next, the dynamic viscosity, electrophoretic mobility (EM) and conductivity of these solutions were determined. Solution dynamic viscosity (*n* = 3) was measured using a cone and plate rheometer under dynamic shear (TA instruments, New Castle, DE). Briefly, 2 ml of sample was placed on the instrument stage maintained at 22°C and a conical platen was lowered on top, ensuring full coverage. Next, a dynamic frequency sweep was conducted from 0.01–158 Hz and viscosity was calculated as the slope of the stress:strain rate curve. Electrophoretic mobility (*n* = 3) and conductivity (*n* = 3) were measured using the Zetasizer Nano ZS (Malvern) with disposable cuvettes (Malvern DTS1070) following the manufacturer’s recommended protocol.

Next, the feasibility of electrospinning a fibrous gelatin mesh using the gelatin-solvent combinations described above were tested with a custom electrospinning device (Subramony et al., 2014). The gelatin solutions were loaded into a 5 ml syringe (Becton Dickinson, Franklin Lakes, NJ) fitted with a blunt-tipped 18-gauge stainless steel needle (Becton Dickinson). Once cooled to room temperature (∼22°C), the loaded syringe was mounted onto a syringe pump (Harvard Apparatus, Holliston, MA) and electrospun at a set flow rate, voltage, relative humidity, and needle distance from a collection drum in a custom chamber. The drum was rotated at 500 rpm to promote even distribution of the collected fibers and uniform mesh thickness. The voltage and flow rate were optimized by identifying stable electrospins while adjusting the voltage from 5–25 kV and the flow rate between 0.2–5.0 ml/h. The chamber relative humidity was controlled at 40% and the travel distance between the needle and drum was fixed at 15 cm. The spins were considered stable when a fiber ejection persisted for greater than 15 min without user intervention.

### Mesh characterization

Post fabrication mesh fiber morphology, chemical composition, and mechanical properties under tensile loading were determined. The electrospun meshes were first cored with a biopsy punch (Sklar Surgical Instruments, West Chester, PA) to obtain 10 mm discs. Fiber diameter (*n* = 50 fibers/group) and morphology (*n* = 5) were characterized by scanning electron microscopy (SEM, Zeiss Sigma VP, 2 keV, Carl Zeiss AG, Oberkochen, Germany). Prior to imaging, the samples were sputter-coated with gold-palladium using a 108 Auto Manual sputter coater (20 s, ∼10 nm, Cressington Scientific, Watford, United Kingdom) to ensure sample conductivity. In addition to visual assessment of fiber continuity and morphology, fiber diameter was measured at ×10,000 magnification using ImageJ (National Institutes of Health, Bethesda, MD, *n* = 5 images per group, *n* = 10 fibers per image), as previously described (Lee et al., 2017).

Mesh chemical composition (*n* = 3) was confirmed by Fourier transform infrared spectroscopy in attenuated total reflectance mode (FTIR-ATR, Spectrum 100, Perkin Elmer, Waltham, MA). Spectra were collected at 100 scans with a spectral resolution of 4 cm^−1^. Characteristic collagen peaks (Amide A, 3,306 cm^−1^; Amide B, 3,076 cm^−1^; Amide I, 1,646 cm^−1^; Amide II, 1,520 cm^−1^; Amide III, 1,234 cm^−1^) (Muyonga et al., 2004; Nagai et al., 2010; Di Foggia et al., 2011; Cebi et al., 2016; Xu et al., 2017) were monitored in relation to solvent types, both pre- and post- electrospinning.

Sample mechanical properties (*n* = 6) were determined in tension according to the ASTM International standard test method D638-14 (Lu et al., 2005; Moffat et al., 2009). Briefly, 4 × 1 cm sections of mesh ranging from 0.1–0.3 mm thick were loaded onto a microtester device (Instron, Model 5,848, 10 N load cell, Norwood, MA), targeting a final gauge length of 2–3 cm. The samples were tested to failure at a strain rate of 5 mm/min. Young’s modulus, yield strength, tensile strength, and ductility were calculated using a custom MATLAB code. Briefly, Young’s modulus was measured as the slope of the linear region of the stress strain curve (0.2% strain offset) immediately following the toe region and prior to the onset of plastic deformation. The yield strength was calculated at the point where the stress strain curve was no longer linear, indicating plastic deformation. The tensile strength was calculated at the maximum stress measured in the curve the prior to sample failure. Lastly, the ductility was calculated by measuring the % elongation at the time of sample failure.

### Mesh chemical crosslinking optimization

The electrospun gelatin meshes were chemically crosslinked using glutaraldehyde (GTA) and the effect of time, sample surface area, and ambient conditions were determined. To evaluate the effect of crosslinking time, as-fabricated meshes (6 × 6 cm) were secured in a custom bracket and placed inside of a beaker (2 L) containing 15 ml of GTA solution (Sigma G6403) and loosely covered (Figure 2A). The distance between the mesh-bracket system and the GTA bath was fixed at 6 cm. The mesh was exposed to the GTA vapors from the bath in a fume hood (Air Flow = 80 CFM) for 0.5, 1.0, 1.5, and 6 h to allow for crosslinking.

**FIGURE 3:**
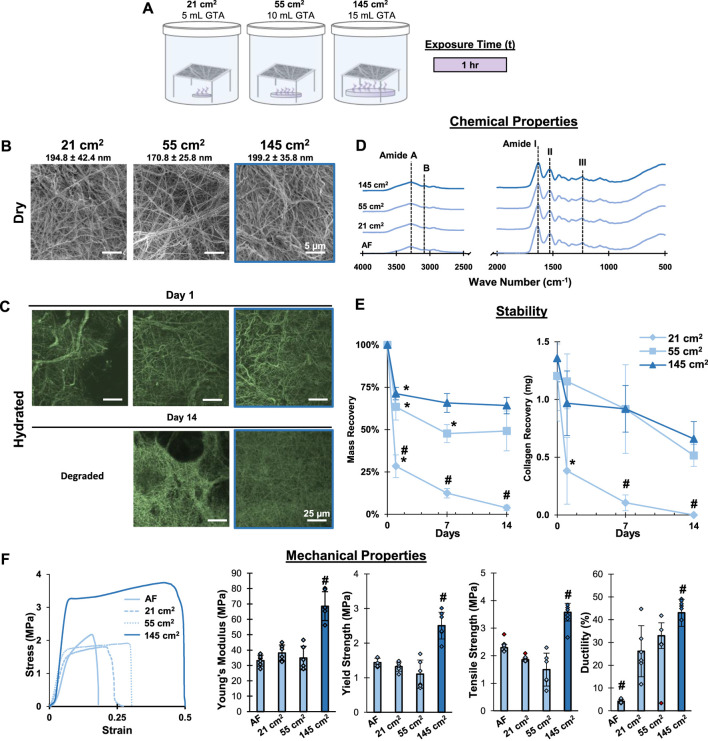
**Mesh Fabrication: Crosslinking Surface Area.** Crosslinking with the 145 cm^2^ surface are a bath for 1 hour stabilizes gelatin meshes and enhances tensile properties without changing fiber morphology, chemistry, or causing pits/voids in the mesh surface. **(A)** Schematic of the crosslinking procedure. **(B)** Dry morphology (SEM, 2,500x) and **(C)** hydrated morphology (CRM, 100x) of meshes after crosslinking. **(D)** Chemical composition (FTIR-ATR). **(E)** Mesh stability by mass recovery and collagen recovery after incubation at 37°C in cell culture medium (14 days, *n* = 5). **(F)** Mesh tensile properties with representative stress-strain curves (*n* = 10). Red markers represent outliers. AF=As fabricated. #=*p* < 0.05 between groups. *=*p* < 0.05 overtime.

After identifying the optimal crosslink duration, the effect of surface area of the GTA bath was determined at 21 cm^2^, 55 cm^2^, and 145 cm^2^. A similar set-up was followed as described above (Figure 3A), except, for the 21 and 55 cm^2^ groups, GTA was poured into small and medium sized dishes with desired surface areas instead of the bottom of the glass beaker (glass beaker surface area was 145 cm^2^). All meshes were then exposed to GTA baths for the optimal duration as determined above.

**FIGURE 4:**
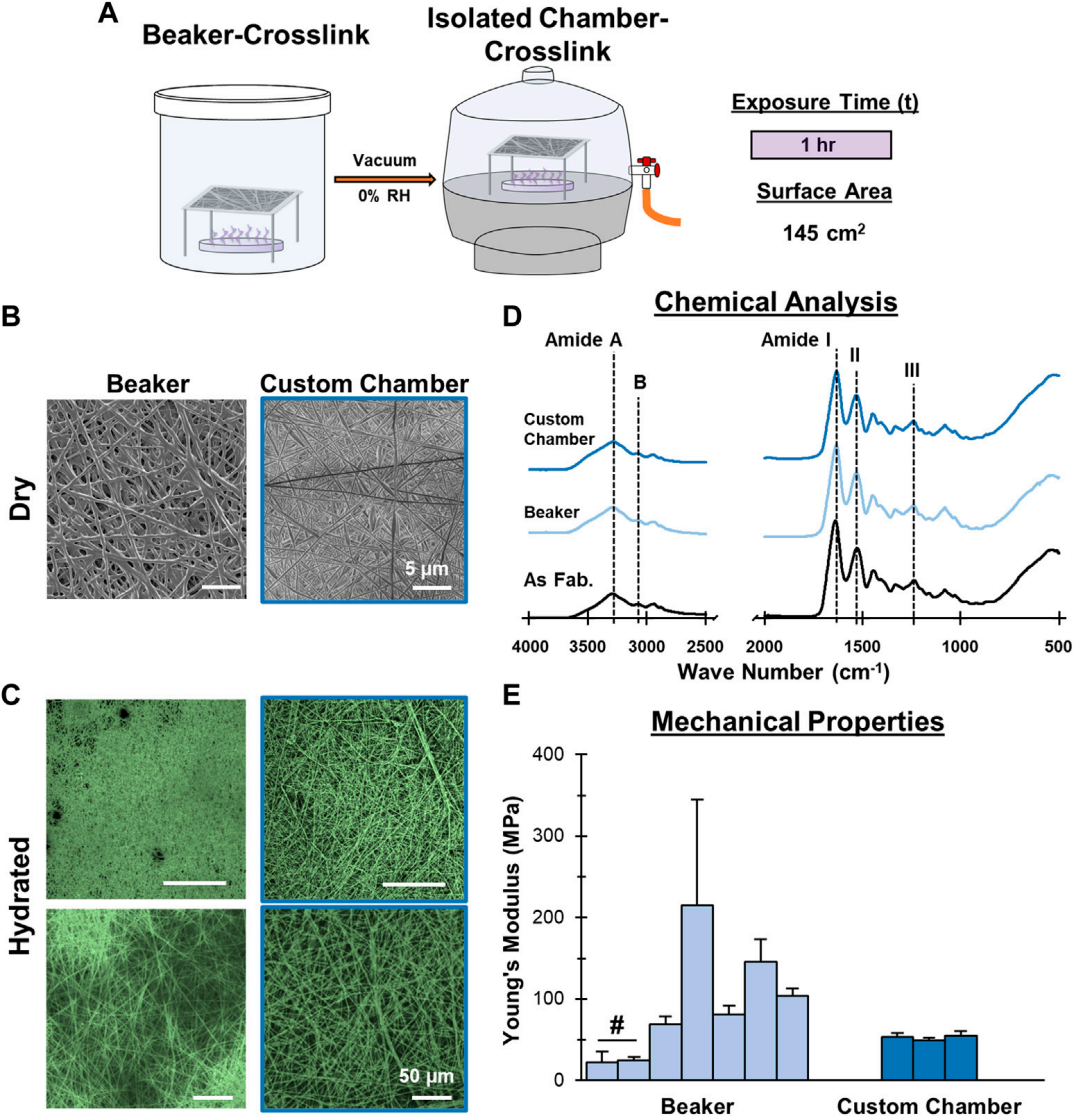
**Mesh Fabrication: Crosslinking Environmental Conditions**. Crosslinking inside a vacuum sealed chamber minimizes batch-to-batch variability. **(A)** Schematic of the crosslinking procedure. **(B)** Dry morphology (SEM, 2,500x) and **(C)** hydrated morphology (CRM, 100x, 300x) of meshes after crosslinking. **(D)** Chemical composition (FTIR-ATR). **(E)** Young’s Moduli after crosslinking by beaker (7 batches) and the custom chamber (3 batches) (*n* = 10). #=*p* < 0.05.

Lastly, to standardize ambient effects on crosslinking, gelatin meshes were chemically crosslinked by glutaraldehyde vapor deposition in a custom chamber (Figure 4A). Briefly, a 15 ml glutaraldehyde bath was placed in the custom chamber. A gelatin mesh (6 × 6 cm) was secured in a custom bracket and suspended 6 cm above the GTA bath with optimized surface area. The custom chamber was then sealed by applying a vacuum. After 3 min, the valve was closed and the chamber was isolated, allowing it to sit under vacuum for the optimal duration identified above. After half of the “optimal duration” had passed, the vacuum was slowly released to prevent GTA aerosolization and the mesh was flipped over. The vacuum was then reapplied as described above, allowing the mesh to sit under vacuum for the remainder of the prescribed time. Sample chemistry, structural and mechanical properties were compared between three batches of as-crosslinked meshes.

Optimal crosslinking parameters were determined by evaluating the impact the crosslink process had on mesh stability, fiber morphology, chemical composition, and mesh tensile properties. More specifically, the crosslinking conditions should not alter the mesh fiber diameter, morphology and chemical composition from that observed in the as-fabricated meshes. In addition, the crosslinking process should enhance mesh mechanical properties and promote mesh stability by preventing loss of no more than 50% of its mass after incubation under cell culture conditions for up to 14 days. Finally, optimal crosslinking parameters should produce meshes with consistent chemical and physical properties without significant batch to batch variation.

### Characterization of crosslinked meshes

Mesh fiber diameter, morphology, tensile properties, chemical composition and stability were evaluated as a function of crosslinking conditions. Fiber diameter and morphology were analyzed by SEM as described above. In addition, hydrated mesh morphology and stability (*n* = 2) were visualized by scanning confocal reflectance microscopy (CRM) (Paddock, 2002; Yang and Kaufman, 2009) on an inverted fluorescent microscope (Olympus Fluoview IX70 Confocal Microscope). The microscope was outfitted with ×20 (air) and ×40 (oil) objectives. Samples were illuminated (488 nm) and the reflected light was detected by a photomultiplier tube. Note that all meshes were compared to “0-h” group, which consists of meshes that had not undergone any crosslinking and were imaged in 100% ethanol to prevent dissolution.

Mesh mechanical properties (*n* = 6) and chemical composition (*n* = 3) were determined as described above. Mesh stability was evaluated qualitatively, through CRM imaging, and quantitatively by recording mesh mass and gelatin recovery after 14 days of incubation in Dulbecco’s Modified Eagle’s Medium (DMEM, Corning) supplemented with 10% fetal bovine serum (FBS, Atlanta Biologicals, Atlanta, GA), and 1% penicillin-streptomycin (P/S, 10,000 U/mL penicillin, 10 mg/ml streptomycin). Briefly, the mass of 1 cm^2^ squares of mesh (*n* = 5) was measured at 0, 1, 7, and 14 days. After incubation, the samples were rinsed three times with deionized water and then dried using a CentriVap (Labconco, Kansas City, MO). Sample dry mass was recorded and mass recovery was calculated relative to the sample’s starting mass. Mesh recovery was further determined using a hydroxyproline assay (Reddy and Enwemeka, 1996). Briefly, after dehydration, samples were digested for 18 h at 65°C with papain (8.3 activity units/mL) in 0.1 M sodium acetate, 10 mM cysteine-HCl, and 50 mM ethylenediaminetetraacetate. Next, samples were hydrolyzed by 2 N sodium hydroxide and incubated in Chloramine-T and Ehrlich’s reagent. Absorbance was measured at 555 nm (TECAN SpectraFluor Plus) and gelatin content was calculated by correlating sample absorbance to a collagen standard curve.

### Biocompatibility—Cells and cell culture

Biocompatibility of the gelatin mesh was assessed *in vitro* using both primary stromal cell culture and an established cell line. All cell culture was conducted in fully supplemented (F/S) media consisting of DMEM supplemented with 10% fetal bovine serum (FBS, Atlanta Biologicals, Atlanta, GA), and 1% penicillin-streptomycin (P/S, 10,000 U/mL penicillin, 10 mg/ml streptomycin). Crosslinked gelatin meshes (1 × 0.5 cm) were sterilized with 70% ethanol for 15 min, followed by rinsing in phosphate buffered saline (PBS, 2x, 5 min). After the second PBS rinse, meshes were pre-soaked in F/S media for 2 h to prepare meshes for cell attachment. Bone marrow derived stromal cells (MSC, Lonza, passage 4) (Bogdanowicz and Lu, 2017) or human osteoblast-like cells (HTB-85 Saos-2, ATCC, passage 8) were seeded onto meshes by dispensing 5 µL of concentrated cell stock suspension (1 M cells/ml) onto the mesh surface, targeting a seeding density of 50,000 cells/cm^2^. Cells were allowed to attach for 20 min, after which 1 ml of F/S media was added and the samples were cultured at 37°C and 5% CO_2_ for up to 28 days. Media was refreshed three times per week. After 6 days of culture, all groups were treated with 3 mM β-glycerophosphate, which was refreshed at each feed.

### Cell response

Cell viability (*n* = 2) on the gelatin mesh was determined by Live/Dead staining (Molecular Probes, Eugene, OR) according to the manufacturer’s protocol. Briefly, the samples were labeled with Live/Dead reagent warmed to 37°C for 2 minutes followed by rinsing in PBS. Next, all samples were imaged by confocal fluorescence microscopy (Olympus Fluoview IX70) at excitation/emission wavelengths of 473/519 nm for live imaging and 559/635 nm for dead imaging. All images are shown as an overlay of the live and dead channels.

Following cell culture, cells were lysed by rinsing the meshes with PBS and freezing them in 500 µL of 0.1% v/v Triton-X solution (Sigma-Aldrich). After one freeze/thaw cycle, samples were ultrasonicated for 10 s at 5 W (Misonix XL-2000, Farmingdale, NY) to promote cell lysis.

Cell proliferation (*n* = 5) was assessed using a Quanti-iT™ PicoGreen^®^ dsDNA assay kit (Molecular Probes) according to the manufacturer’s protocol. Fluorescence was measured using a microplate reader (SpectraFluor Plus, Tecan, Research Triangle Park, NC) at excitation and emission wavelengths of 485 and 535 nm, respectively. Total cell number per sample was determined by correlating measured fluorescence intensity to a DNA standard curve and using a conversion factor of 7.7 pg DNA/cell (Jiang et al., 2005).

Collagen content (*n* = 5) was assessed by using a hydroxyproline assay (Reddy and Enwemeka, 1996). Briefly, the sample were first desiccated for 12 h in a CentriVap Concentrator to remove all liquid and digested for 18 h at 65°C with papain (8.3 activity units/mL) in 0.1 M sodium acetate, 10 mM cysteine-HCl, and 50 mM ethylenediaminetetraacetate. Collagen was hydrolyzed by mixing aliquots of the digested mesh with 2 N sodium hydroxide and heating to 250°C for 25 min. The resulting sample hydrolysate was oxidized at room temperature (25°C) for 25 min with buffered Chloramine-T reagent. Ehrlich’s reagent (15% p-dimethylaminobenzaldehyde in 2:1 isopropanol/perchloric acid) was then added and absorbance was measured at 555 nm with a microplate reader. Hydroxyproline content was determined by correlating measured optical intensity to a bovine collagen I (Biocolor, Carrickfergus, United Kingdom) standard curve.

Matrix mineralization potential, as reflected in ALP activity (*n* = 5), was assessed using a colorimetric assay based on dephosphorylation of p-nitrophenyl phosphate (pNP-PO4) to p-nitrophenol (pNP) (Lu et al., 2003). An aliquot of lysed sample was mixed with pNP-PO_4_ solution (Sigma-Aldrich) at a 1:1 v/v ratio and incubated at 37°C for up to 30 min. Absorbance was measured using a microplate reader at 405 nm after 5 min for Saos-2 cultures and after 30 min for MSC cultures. Sample ALP activity was determined by correlating measured optical intensity to a pNP standard curve and normalized to cell count and the time the sample was allowed to react.

Mineralization was further ascertained by energy dispersive x-ray analysis (EDS, XFlash^®^ 6–30, Espirit 2.1 software, Bruker, Billerica, MA). Briefly, quantitative spectra were collected at ×10kV, ×500 magnification using the automated “fast” acquisition software settings. Spectra was then analyzed using the Espirit 2.1 software and Ca and P content (*n* = 3) was calculated in ppm relative to all elements detected. Mineral distribution (*n* = 2) was visualized by Alizarin Red and Von Kossa staining. Briefly, following incubation, the samples were rinsed 2x with PBS and then fixed in 10% neutral buffered formalin containing 1% cetylpyridinium chloride for 1 h at room temperature. After fixing, samples were rinsed 4x with deionized water and soaked overnight in 5% (w/v) polyvinyl alcohol (PVA). After soaking, samples were embedded in a block of PVA, frozen, and sectioned with a cryotome (10 μm, CM3050 S, Leica Biosystems, Wetzlar, Germany). After drying overnight, sections were cleared of PVA using deionized water and stained for calcium and phosphate content. To visualize Ca, meshes were stained with 2% Alizarin Red for 3 min, rinsed 4x with deionized water and allowed to airdry prior to imaging. To visualize phosphate, sections were covered with 5% AgNO_3_ and exposed to UV light (365 nm) for 25 min. Following UV exposure, samples were rinsed 4x with deionized water and allowed to air dry. Once dry, all samples were imaged at 10x and 20x under light microscopy with a full spectrum color camera (Zeiss Axiovert 25, Oberkochen, Germany). During imaging, all acquisition settings remained constant to avoid biasing analysis.

### Reagents

All reagents were purchased from Sigma-Aldrich unless otherwise noted.

### Statistical analysis

All results are presented as mean ± standard deviation. The number of sample replicates are reported as “n.” Multiple trials were conducted to ensure reproducibility and only data from representative trials are shown. One-way and two-way analysis of variance (ANOVA) were performed to evaluate statistically significance between sample groups and temporally, when appropriate. A Tukey–Kramer post-hoc test was used for all pair-wise comparisons (*p* < 0.05). All statistical analyses were performed using JMP IN statistical software (SAS Institute, Cary, NC, United States).

## Results and discussion

### Mesh fabrication: Gelatin-solvent interactions

The focus of this study was to identify ideal fabrication and crosslinking conditions for collagen-based materials prepared by electrospinning. We began by first optimizing the use of the “green” and biocompatible electrospinning solvent, acetic acid (AA). To achieve this, we evaluated the solution viscosity, electrophoretic mobility, and conductivity as a function of gelatin concentration in both 50% and glacial AA. For comparison, the traditional solvent trifluoroethanol (TFE) was used as a positive control (Figure 1). Both glacial and 50% AA were investigated because diluted AA can ionize, which impacts the solvency and solution conductivity (Angammana and Jayaram, 2011).

In TFE, gelatin solutions were slightly cloudy above 7% w/v, while this became evident at 10% w/v gelatin in 50% AA, indicating a decreasing solubility with increasing gelatin concentration. After dissolution, it was also observed that gelatin solutions above 7% in glacial AA began to form solid gels, suggesting poor solvency and that glacial AA is not suitable for electrospinning at higher gelatin concentrations. As expected, solution viscosity increased significantly with gelatin concentration for all solvents tested. The 5% and 7% gelatin in TFE solutions had viscosities of 12.8 and 35.3 cP, respectively, while 20 and 30% w/v gelatin in 50% AA exhibited viscosities of 333.2 and 567.6 cP, respectively. Because gelatin formed particulates at 13% w/v in TFE, higher concentrations were not evaluated. As well, because gelatin gelled in glacial AA above 7% w/v, viscosity, mobility, and conductivity were not measured. Typically, electrospun solutions with viscosity greater than 100 cP are suitable for electrospinning because it indicates there are sufficient cohesive forces between solute particles to promote chain entanglement and that the solution can resist the applied electrostatic forces until a critical potential is achieved. At this critical potential, the solute will eject from the solution, forming a steady release of fine, smooth fibers (Fong et al., 1999; Ginestra et al., 2016; Amariei et al., 2017). While it is possible to electrospin gelatin at lower viscosities, the fiber ejection is often less stable due to the lower cohesive forces, resulting in the need for frequent user intervention during fabrication and increased variability in mesh morphology (Zeng et al., 2003; Nezarati et al., 2013).

Next, taking a closer look at the electrochemical properties, the mobility of gelatin in each sample was measured (Figure 1B). For all TFE samples, mobility measurements were below the limit of detection for the instrument, suggesting there were not enough particles with sufficient charge and/or the solution’s ionic strength was too low be conductive. Interestingly, in 50% AA samples, the maximum mobility (0.28 µm * cm/Vs.) was observed in the 7% w/v gelatin solution, while mobility of 0.09 µm * cm/Vs. and 0.04 µm * cm/Vs. were measured in the 20% and 30% solutions, respectively. Since the mobility represents the ability of a solute to move in solution in response to an applied electric field (Davis and Cohn, 1939), it is an important factor for solution spinnability, which relies on polymer migration within the solvent prior to ejection. Thus, it is likely that a stable electrospin will result when solutions exhibit a moderate to low mobility. In this scenario, gelatin particles will migrate through solution quickly enough to eject, but slowly enough to promote polymer entanglement, which is critical for steady fiber ejection (Haider et al., 2018; Xue et al., 2019). If the mobility is too low, particles may fail to migrate and eject, while conversely, if the mobility is high, particles will migrate too quickly in solution, resulting in ejection before polymer entanglement can occur causing samples to electrospray as fine particles. In this study, the 20% and 30% w/v gelatin in 50% AA resulted in the most stable spins, as these solutions exhibited the lowest, but still non-zero mobility among all the samples.

In terms of conductivity, the TFE, 50% AA, and glacial AA neat solutions (no gelatin) had conductivities of 0.00 mS/cm, 0.98 mS/cm, and 0.05 mS/cm, respectively (Figure 1B). The TFE solutions exhibited an increase in conductivity with gelatin concentration, reaching a maximum conductivity of 36.5 μS/cm at 13% gelatin. As expected, conductivity of AA significantly increased when mixed with water. This is because diluting AA results in increased ionization, creating conductive acetate and hydronium ions. The conductivity for glacial AA increased slightly to 0.10 mS/cm with the addition of 7% gelatin, but was not measurable at higher concentrations due to the gelation of the samples. For 50% AA solutions, the conductivity increased with gelatin concentration, reaching 3.03 mS/cm in the 30% w/v gelatin sample, which is significantly greater than all TFE and glacial AA solutions tested. Conductivity is a vital characteristic for establishing a stable, reproducible electrospin (Uyar and Besenbacher, 2008; Angammana and Jayaram, 2011), as a sufficient number of ions must be present to permit the transfer of charge from the applied electric force that ultimately induce particle migration, entanglement, and ejection into continuous fibers. Zhang et al. (2005b) observed that increasing solution conductivity resulted in significant decreases in polyvinyl alcohol fiber diameter but, once above 2.75 mS/cm, resulted in bead formation along the polymer strand. Fong et al. (1999) reported that below a certain charge density threshold, which is analogous to solution conductivity, fiber ejection was not continuous and that bead formation would also occur in polyethylene oxide fibers, suggesting a minimum conductivity is also critical. These observations suggest that 50% AA is the best suited solvent for electrospinning, as it is electrically conductive and can support the building of the charge that is critical for inducing solute:solvent instability and causing fiber ejection.

Electrospinning stability relies on a balance between polymer chain mobility and entanglement while in the spin solution. Sufficient polymer entanglement is necessary to support steady polymer ejection while a sufficient polymer migration is necessary for solute movement and ejection. Here, we evaluated the solution viscosity, mobility and conductivity, to identify ideal solution properties. While mobility is a direct measure of particle movement the solution viscosity and conductivity are primarily indicative of particle entanglement and migration, respectively. Based on our analysis, 20% and 30% gelatin in 50% AA are most likely to result in stable electrospins due to their high conductivity, high viscosity, and moderate to low mobility. These properties in conjunction, will support polymer chain entanglement and migration, allowing for a steady fiber ejection. Interestingly, it was observed that gelatin in TFE did not follow this pattern, yet gelatin has previously been electrospun using TFE (Huang et al., 2004; Zhang et al., 2005a; Choktaweesap et al., 2007; Chuang, 2015). This is possible because, while each of these solution properties independently may not be ideal for electrospinning, they collectively can still support fiber formation, albeit with less stable fiber ejection. The instability is a direct consequence of the solution’s low conductivity and viscosity, which result in insufficient solution charging to support mobility and evoke polymer chain entanglement, respectively. However, despite this, fiber ejection is still achieved due to the lower mobility of the polymer particles. This is because the lower mobility still allows particles to entangle and migrate, offsetting the low conductivity and viscosity. Conversely, if these solutions exhibited large mobility, gelatin particles would rapidly move and eject during the spin, resulting in electrospraying. Thus, even with sub-optimal solution characteristics, gelatin can be electrospun in TFE, albeit with less stable ejection that requires more user intervention and increased variability. In contrast to TFE, the 50% AA gelatin solutions had significantly greater viscosity, mobility, and conductivity. These three solution properties worked in conjunction to allow for sufficient polymer chain entanglement and mobility, which should result in a steady ejection of polymer fibers with minimal user intervention required during fabrication.

### Mesh fabrication: Electrospinning parameters

After characterizing solution properties, spinnability was evaluated at flow rates ranging from 0.25–5.0 ml/h and applied voltages ranging from 5–25 kV. These test parameters were chosen because they are reported to be stable electrospins (Moffat et al., 2009; Mosher et al., 2021). An electrospin was considered stable when a single, steady fiber ejection persisted without the need of user intervention for >15 min. Once stable spin conditions were identified, the resulting fibers were evaluated to ensure they retain the chemical properties of the source gelatin and are comprised of smooth fibers with physiologically relevant diameters and mechanical properties.

It was observed that gelatin prepared in TFE at 5% and 7% w/v produced stable spins after optimization of melt flow rate and applied voltage (Figures 1C,D), albeit with periodic user intervention and maintenance. When mixed in 50% AA, gelatin failed to electrospin at concentrations below 20% w/v. At 30% w/v, fibers appeared glassy and ribbon like, losing the cylindrical morphology (Figures 1C,D). These initial observations suggest that 7% and 30% w/v gelatin may be the upper limit for producing fibrous meshes in TFE and 50% AA, respectively. As shown in Figure 1C, all stable spins resulted in fibers with diameters averaging less than 300 nm, with the exception of 30% w/v gelatin in 50% AA. As expected, fiber diameter increased with gelatin concentration regardless of solvent tested. The greater solute per unit volume and higher viscosity that is associated with increasing gelatin concentration result in larger fibers along with decreased fiber stretching during ejection (Fridrikh et al., 2003; Zeng et al., 2003).

Given that the 20% w/v gelatin in 50% AA and 7% w/v gelatin in TFE were stable spins and had comparable fiber diameters, these two conditions were selected for further characterization (Figures 1E,F). FTIR-ATR analysis identified the characteristic gelatin or collagen peaks, including amide A (N-H stretch, 3,306 cm^−1^), amide B (C-H stretch, 3,076 cm^−1^), amide I (C = O and C-N stretch, 1,646 cm^−1^), amide II (N-H bend and C-N stretch, 1,520 cm^−1^), and amide III (N-H bend, C-N stretch, C-C and C = O in-plane bend, 1,234 cm^−1^) (Muyonga et al., 2004; Nagai et al., 2010; Di Foggia et al., 2011; Cebi et al., 2016; Xu et al., 2017) All were evident in spectra for the source gelatin and in meshes prepared in TFE and AA, indicating that no chemical modifications occurred due to reacting with the solvent nor the electrospinning process. For mechanical properties, both the TFE positive control and 50% AA group showed comparable Young’s Moduli of approximately 30MPa, which is on the order of most biological tissues (Graham et al., 2004; Akhtar et al., 2011). Yield strength, ultimate tensile stress, and ductility were observed to be higher in the 50% AA mesh. The tensile properties of various hydrated collagen-based tissues have been reported to be in the range of 2–27 MPa (Eleswarapu et al., 2011), suggesting that the 50% AA mesh may be more representative of the softer tissues tested, while the TFE mesh was even weaker when tested wet under tension. The enhanced mechanical properties found in the 50% AA meshes may be due to carboxymethylation and subsequent crosslinking that can occur in AA, which is known to increase mesh mechanical properties (Yamauchi et al., 1988; Gómez-guillœn and Montero, 2001). Additionally, differences in solvent evaporation rate may impact the mechanical properties of the fibers by altering the polypeptide chain organization and ultimate crystallinity (Coppola et al., 2012). More specifically, because AA is less volatile than TFE, it will evaporate slower during the spinning process. This slower evaporation can allow polypeptide chains to rearrange to lower energy states, impacting sample crystallinity and enhancing mechanical properties. Lastly, SEM micrographs (Figure 1D, black-boxed and blue-boxed micrographs) showed that both meshes contained smooth, randomly oriented, nanofibers that appeared morphologically identical to many ECM tissues. Thus, gelatin was successfully electrospun in 50% AA with comparable modulus and morphology to traditional electrospinning methods (TFE). Moreover, the meshes were structurally and compositionally similar to natural ECM, supporting the use of this substrate as an ECM analog.

### Mesh fabrication: Chemical crosslinking

Electrospun gelatin or collagen meshes require crosslinking to prevent dissolution and loss of structure in aqueous environments such as cell culture media. Therefore, the next part of this study centered on developing an optimized chemical crosslinking strategy that is reproducible and retains both morphological and chemical features of the as-fabricated mesh. Specifically, GTA is used to crosslink the meshes due to the ability of its dialdehyde groups to covalently bind primary amines present along the amino acids that make up gelatin, creating strong chemical crosslinks. To this end, the effects of crosslinking time, GTA bath surface area, and the impact of environmental conditions, including air flow and relative humidity, were evaluated. During each step of the optimization, the resulting mesh stability, fiber morphology, chemical composition, and tensile properties were analyzed. Along with the as-expected increase in mechanical properties due to crosslinking, the optimal crosslinking conditions were defined as the production of physically stable meshes in dry and wet conditions with unaltered fiber morphology.

To evaluate the impact of crosslinking duration on the gelatin mesh, samples were exposed to GTA vapors for 0.5, 1.0, 1.5, and 6 h in a loosely-capped 2-L beaker containing 15 ml of GTA (Figure 2). A noticeable change in mesh morphology was observed at 1.5 h (Figure 2B, SEM). The fibers began to appear less cylindrical and woven and took on a flat, mat-like appearance. This became more apparent after 6 h, as the mesh fiber morphology and apparent pore size, which are critical features for recapitulating the native ECM, were starting to be irreversibly changed. Moreover, fibers began to merge together at 1.5 h, resulting in increases in the apparent fiber diameter. Furthermore, with increasing duration, meshes were becoming noticeably more brittle, making them difficult to handle.

Next, mesh stability was assessed in cell culture media, focusing on maintaining mesh morphology and mass over time. After 2 weeks, distinct fibers were retained in all groups except the 0.5-h group, where numerous pits and voids were observed, indicating 30 min crosslinking is insufficient for stabilizing gelatin *in vitro* (Figure 2C). In the 6-h group, large beaded structures were observed on the mesh surface. These beads are likely the result of GTA droplets, instead of fine vapors, becoming airborne during long crosslinking periods (beyond 1.5 h). These drops then contact the mesh, merging the nano-fibers into larger structures disrupting fiber morphology. Thus, 0.5-h was insufficient for stabilizing the mesh, while 1.5 and 6 h were too long since abundant bead formation was observed. Furthermore, the 1-h group retained the most similar morphology to the as-fabricated day 0 mesh, which was imaged in 100% ethanol to preserve the mesh structure for comparison.

Meshes crosslinked for 6 h showed no significant change in mass, while the 1-h and 1.5-h groups showed 67% and 71% mass recovery after day 1, respectively (Figure 2E). Mesh mass stabilized thereafter for both groups, suggesting that the initial loss may be attributed to non-crosslinked gelatin. The lowest mass recovery (17%) at day 1 was found in the 0.5-h group, with significantly lower mass recovery over time, indicating the mesh was not sufficiently crosslinked. Along with mass recovery, gelatin recovery was also characterized (Figure 2E), which correlated well with the trends observed in mass recovery, indicating that the changes in mass recorded over time were due to loss of gelatin. In addition, this data further corroborated that meshes crosslinked for 0.5 h are unstable, while 1 and 1.5-h crosslinking demonstrated stability after day 1 and meshes crosslinked for 6 h remained stable through day 14. Reviewing the IR spectra, no discernible differences between as-fabricated and crosslinked meshes were observed, regardless of the duration (Figure 2D). This suggests that the chemical bonds formed during the crosslinking procedure may not be distinguishable by this method, but also confirms that other unintended modifications are not occurring during the crosslinking process. Following IR analysis, the tensile properties of the meshes, including Young’s Modulus, yield strength, tensile strength, and ductility, were evaluated. As expected, mechanical properties increased with crosslinking time, with the highest value measured after 6 h. Notably, however, greater variability was found in the mechanical properties of the 6 h group, which is likely attributed to the increased mesh brittleness that is a result of overexposure to GTA.

Collectively these observations indicate that the ideal crosslinking duration is 1-h, which is sufficient for stabilizing the gelatin meshes without compromising fiber morphology and mechanical properties. Crosslinking for 0.5 h resulted in significant mass loss, while crosslinking for 1.5 and 6 h led to loss of fiber morphology and the mesh fibrous network. Thus, for all subsequent crosslinking optimization, 1 h was chosen as the ideal duration.

After determining an optimal crosslinking duration, the impact that the GTA bath surface area had on crosslinking was investigated. Increasing bath surface area increases vapor formation, thus impacting the crosslinking process. Thus, an optimal bath surface area will produce sufficient vapors to stabilize the meshes without altering their chemical or morphological features. To evaluate this, gelatin meshes were suspended over baths with surface areas of 21 cm^2^, 55 cm^2^, and 145 cm^2^ and crosslinked for 1.0 h (Figure 3). Following crosslinking, no significant changes in fiber diameter nor fiber arrangement and morphology were observed among the different groups (Figure 3B). Imaging by CRM found loss in fiber morphology for the 21 cm^2^ group after just 1 day of incubation in culture media (Figure 3C). Intact fibrous structures were observed in the 55 and 145 cm^2^ setups by CRM, however, pitting and voids were found in the 55 cm^2^ after 14 days of incubation. Mass recovery analysis found an 18% mass recovery for the 21 cm^2^ after 1 day of incubation, while mass recovery was 62% and 71% for the 55 and 145 cm^2^, respectively (Figure 3E). By day 14, meshes crosslinked with 21 cm^2^ surface area fully dissolved, while 55 and 145 cm^2^ groups remained relatively stable at 49% and 64% mass recovery, respectively. Similar trends were also observed after analyzing gelatin recovery for these samples using the hydroxy-proline colorimetric assay. Analysis by FTIR-ATR did not show any changes, indicating that chemically, gelatin remained unchanged and that GTA crosslinking was not detected in the IR spectra (Figure 3D). Mechanical properties were enhanced in the 145 cm^2^ group but remained unchanged between the 21 cm^2^, the 45 cm^2^, and the as-fabricated mesh (Figure 3D). Thus, it was determined that 145 cm^2^ surface area was required to fully stabilize the mesh and prevent loss of fiber morphology when crosslinking for 1 h and will be used in further optimization studies.

After identifying optimal duration and surface area parameters, significant variability was still observed in mesh mechanical properties. This variability was found to directly correlate with ambient conditions, such as air flow and humidity, during crosslinking. Suspecting that the loosely-capped beaker exposed the crosslinking to environmental conditions, a custom chamber that isolated the mesh during crosslinking, essentially eliminating humidity and air flow as variables, was developed (Figure 4). To test this new approach, meshes were placed into the custom chamber with a GTA bath with a surface area of 145 cm^2^ and a vacuum was applied. Similar to the optimized beaker approach, the mesh was crosslinked 30 min per side (1 h total). To determine the impact of the chamber, mesh tensile mechanical properties were evaluated (Figure 4E). Three separate batches were crosslinked in the custom chamber under vacuum and exhibited consistent mechanical properties, with Young’s moduli of 53.1, 49.2, and 55.3 MPa. This is contrasted with 7 batches of mesh crosslinked using the optimized conditions in the loosely capped beaker, which had moduli ranging from 21.9–215.1 MPa. In addition to reduced variability in mechanical properties, no unintended changes in chemical composition by FTIR and the fiber morphology were observed. Using the custom chamber improved the reproducibility of mesh crosslinking by eliminating the influence that ambient conditions had on the production of GTA vapors and their subsequent interaction with the gelatin mesh.

### Optimized fabrication protocol and biocompatibility

**FIGURE 5:**
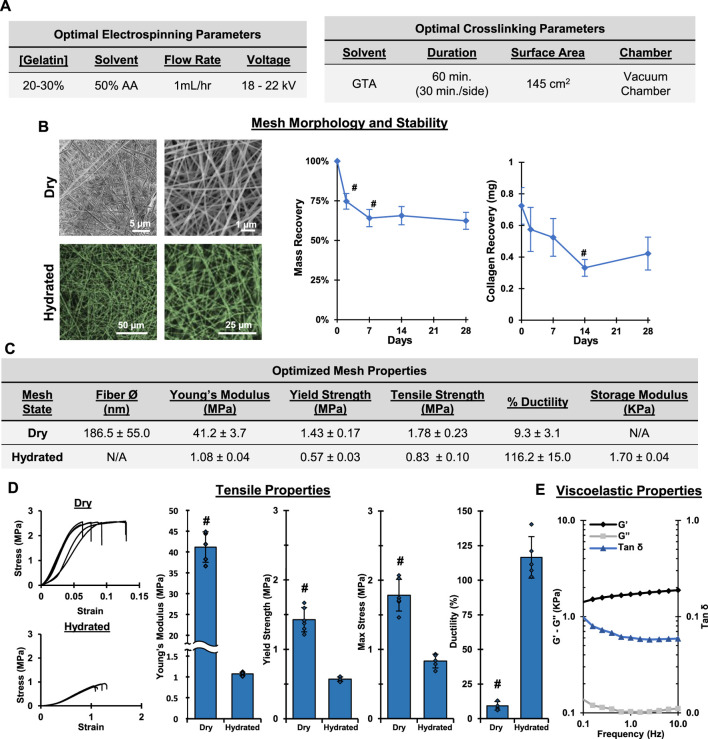
**Optimal Fabrication: Electrospinning and Crosslinking.** Gelatin electrospun in 50% acetic acid and crosslinked for 1 hr in a vacuum chamber results in stable, fibrous meshes with a conserved chemical composition. **(A)** Optimal electrospinning and crosslinking conditions. **(B)** Dry (SEM, 2,500x, 10,000x) and wet morphology (CRM, 100x, 300x) and stability of crosslinked gelatin meshes prepared using optimized parameters. **(C)** Table summarizing properties of optimized mesh. **(D)** Tensile properties after crosslinking and hydrating (*n* = 10). **(E)** Viscoelastic properties of hydrated gelatin meshes (*n* = 3). #=*p* < 0.05.

The processes described above allowed us to determine optimal fabrication and crosslinking conditions for the production of a biomimetic ECM analog using the “green” solvent, AA. Specifically, as shown in Figure 5, the optimal electrospinning conditions are: 20% w/v gelatin in 50% AA at 1 ml/h and 18–22 kV. The optimal setup and conditions for crosslinking includes placing the mesh over a GTA bath (15 ml, 145 cm^2^) for 60 min (30 min per side) under vacuum in a custom chamber. Collectively, these optimal conditions resulted in a stable, fibrous mesh with physiologically relevant mechanical properties that may be used as a biomimetic alternative to current *in vitro* culture platforms (Figure 5). Optimized meshes exhibited Young’s moduli of 41.2 and 1.08 MPa when dry and hydrated, respectively. Furthermore, when hydrated, the meshes were viscoelastic, with a storage modulus of 1.70 kPa. The hydrated meshes were highly elastic and ductile, properties that are similar to many natural ECM (Jiao et al., 2012). It is emphasized that our optimized protocol reproducibly yielded meshes that are chemically and morphologically comparable to collagen matrix and exhibit physiologically relevant physical properties. This is a significant improvement over other commonly utilized approaches for *in vitro* cell culture, including TCPS and synthetic polymer substrates, and demonstrates enhanced reproducibility over similar approaches currently presented in the literature.

**FIGURE 6:**
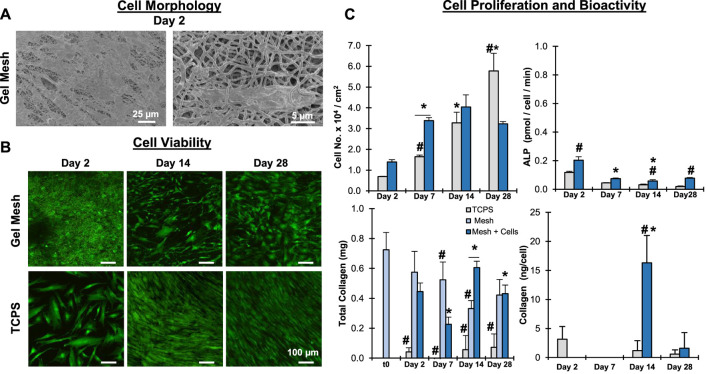
**Mesh Biocompatibility:** **Stem Cells**. Meshes were biocompatible and supported MSC attachment and growth. **(A)** Cell/matric interaction (Day 2, SEM, 500x, 2,500x). **(B)** Cell Viability **(C)** Cell proliferation, bioactivity, and matrix stability (*n* = 5). *= *p* < 0.05 between groups. #=*p* < 0.05 over time.

To assess the biocompatibility of the crosslinked gelatin meshes, mesenchymal stromal cells (MSC) were cultured on the meshes and cell bioactivity and viability were evaluated (Figure 6). Meshes were consider biocompatible if they supported cell attachment, proliferation, and phenotypic response. It was observed that the MSC readily attached to the mesh fibrils (Figure 6A). Furthermore, the cells were observed both atop and beneath mesh fibrils, indicating that they can interact with, respond to, and penetrate the matrix. The stem cells remained viable and proliferated through the course of the study, indicating the mesh did not negatively impact cell health and supports cell proliferation (Figures 6B,C). When normalized to surface area, cell proliferation was greater on the mesh than on TCPS through day 14. At day 14, proliferation on the mesh plateaued, suggesting cell-to-cell contact inhibition may be preventing further expansion. Conversely, on TCPS significant cell proliferation was observed to day 28. This is likely the result of cells spreading to the well-walls and possibly forming cell sheets, which were observed on TCPS but did not occur with mesh samples. Next, minimal ALP activity was observed during the course of the study, suggesting the stem cells are likely maintaining their naïve, undifferentiated state during culture. Collagen content was characterized and it was observed that the MSC did not appear to produce any new matrix, as collagen detected in cellular groups closely correlated with collagen measured in acellular groups. These results clearly demonstrate that gelatin meshes can successfully support stem cell culture prior to induction or differentiation studies.

**FIGURE 7:**
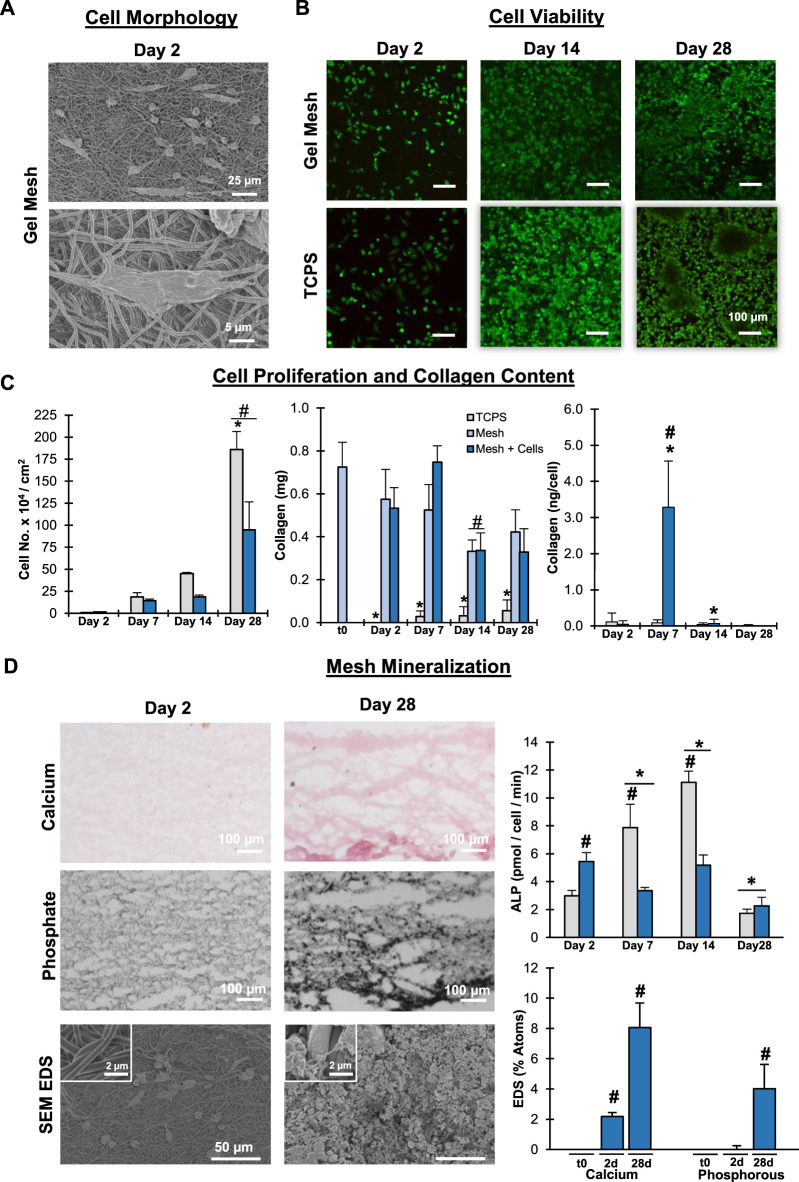
**Mesh Biocompatibility:** **Human Osteoblast-like Cells.** Meshes were biocompatible and supported Saos-2 cell attachment, growth, and matrix mineralization. **(A)** Cell/matrix interactions (day 2, SEM, 500x, 3,000x). **(B)** Cell Viability. **(C)** Cell proliferation and matrix stability (*n* = 5). **(D)** Matrix mineralization qualitatively by Von Kossa and Alizarin Red histological stains and quantitatively by alkaline phosphate activity (*n* = 5) and SEM EDS (SEM, 500x, *n* = 5 regions per mesh). #=*p*< 0.05 between groups, * = *p* < 0.05 over time.

The phenotypic response of a human osteoblast-like cell line on the matrix was also evaluated. Specifically, we were interested in determining if the gelatin mesh can act as a collagen-based platform for cell mediated mineralization. This is of interest because it models one route through which natural tissue mineralization occurs and thus could demonstrate the use of this platform for studying tissue mineralization. Similar to the MSC, the mesh readily supported attachment and proliferation of the osteoblast-like cells (Figure 7). When normalized to surface area, significant proliferation was observed in both mesh and TCPS groups after 28 days of culture. At 28 days, greater proliferation observed in the TCPS can be attributed to the formation of layers of cell sheets as well as cells expanding up the well-walls in the culture plate. Similar to the stem cell data, there was no discernable collagen production when compared to acellular meshes, suggesting the cells may not need to produce additional collagen matrix in the presence of the gelatin mesh. Significantly greater ALP activity was observed from cells cultured on the mesh compared to TCPS at day 2. Alkaline phosphatase is an enzyme critical for catalyzing the conversion of organic phosphate to inorganic phosphates in the body, the rate limiting step that makes up calcium-phosphate minerals in hard tissues (Orimo, 2010). Thus, this elevated activity is not just phenotypically expected, it also demonstrates the mesh can readily act as a base for supporting cell mineralization. Furthermore, it is to be expected that, under normal physiologic conditions, initial increases in ALP activity will be followed by decreases in activity after sufficient amounts of inorganic phosphates are produced. This pattern was observed in the mesh group. Conversely, steadily increasing ALP activity was observed in the TCPS group, which suggests non-physiologic ALP activity that may be related to culturing on TCPS. Mesh mineralization was further corroborated in the SEM/EDS analysis and histological staining (Figure 7D). Mineral nodules were evident on the mesh surface, which was accompanied by significant increases in calcium and phosphorous content on the mesh surface. The increases in CaP on the gelatin meshes may be attributed to cell mediated mineralization. Calcium and phosphate staining positively identified mineral on the mesh surface and through the mesh cross-section after 28 days of culture. Thus, it is clear the gelatin mesh was providing the collagen matrix framework that acts as a substrate for and is functionally similar to the *in vivo* sites for mineralization. This system offers a significant advantage when compared to other systems previously developed for substrate mineralization (Murphy and Mooney, 2002; Nandakumar et al., 2010). Alternative systems often utilize synthetic materials and surface coatings using concentrated simulated body fluid, an ion-rich solution comparable to blood plasma. These non-physiological factors impact the applicability of data collected on these systems when trying to study natural cellular processes. Conversely, in the work presented here, human osteoblast-like cells readily mineralized the surface of the biomimetic gelatin meshes while cultured under physiologically relevant conditions. Thus, the gelatin meshes support cell culture and viability, representing a more biomimetic culture system.

## Conclusion

This study aimed to develop an ECM analog with high fidelity by systematically elucidating optimal fabrication and crosslinking conditions for collagen-based fibers. After optimizing gelatin electrospinning via the use of acetic acid, a “green” and biocompatible solvent, the effects of crosslinking conditions, including duration of exposure, surface area, and ambient conditions were investigated, culminating in optimal parameters for gelatin crosslinking post-fabrication. The resultant mesh is stable in both dry and wet conditions with little batch-to-batch variability, and retains as-fabricated architecture and native chemistry while enhancing mechanical properties. Moreover, the collagen platform supported the maintenance of human stem cells as well as the phenotypic production of a mineralized matrix by osteoblast-like cells. Future studies will explore the potential of this novel ECM analog for elucidating cell-matrix interactions and informing the design of tissue regeneration therapies.

## Data Availability

The raw data supporting the conclusion of this article will be made available by the authors, without undue reservation.
